# Uncertainty Estimation in Medical Image Classification: Systematic Review

**DOI:** 10.2196/36427

**Published:** 2022-08-02

**Authors:** Alexander Kurz, Katja Hauser, Hendrik Alexander Mehrtens, Eva Krieghoff-Henning, Achim Hekler, Jakob Nikolas Kather, Stefan Fröhling, Christof von Kalle, Titus Josef Brinker

**Affiliations:** 1 Digital Biomarkers for Oncology Group German Cancer Research Center (DKFZ) Heidelberg Germany; 2 Department of Medicine III University Hospital RWTH Aachen Aachen Germany; 3 Department of Translational Medical Oncology National Center for Tumor Diseases (NCT) German Cancer Research Center (DKFZ) Heidelberg Germany; 4 Department of Clinical-Translational Sciences Berlin Institute of Health (BIH) Berlin Germany

**Keywords:** uncertainty estimation, network calibration, out-of-distribution detection, medical image classification, deep learning, medical imaging

## Abstract

**Background:**

Deep neural networks are showing impressive results in different medical image classification tasks. However, for real-world applications, there is a need to estimate the network’s uncertainty together with its prediction.

**Objective:**

In this review, we investigate in what form uncertainty estimation has been applied to the task of medical image classification. We also investigate which metrics are used to describe the effectiveness of the applied uncertainty estimation

**Methods:**

Google Scholar, PubMed, IEEE Xplore, and ScienceDirect were screened for peer-reviewed studies, published between 2016 and 2021, that deal with uncertainty estimation in medical image classification. The search terms “uncertainty,” “uncertainty estimation,” “network calibration,” and “out-of-distribution detection” were used in combination with the terms “medical images,” “medical image analysis,” and “medical image classification.”

**Results:**

A total of 22 papers were chosen for detailed analysis through the systematic review process. This paper provides a table for a systematic comparison of the included works with respect to the applied method for estimating the uncertainty.

**Conclusions:**

The applied methods for estimating uncertainties are diverse, but the sampling-based methods Monte-Carlo Dropout and Deep Ensembles are used most frequently. We concluded that future works can investigate the benefits of uncertainty estimation in collaborative settings of artificial intelligence systems and human experts.

**International Registered Report Identifier (IRRID):**

RR2-10.2196/11936

## Introduction

### Overview

Digital image analysis is a helpful tool to support physicians in their clinical decision-making. Originally, digital image analysis was performed by extracting handcrafted features from an input image. These features can be tuned to the underlying data, which means that for a specific disease, only specific features can be looked for in the observed image. With the advent of deep learning, however, a “black box” has been established that can, in the setting of supervised learning, intrinsically learn such features from labeled data. In recent years, deep learning–based methods have vastly outperformed traditional methods that rely on handcrafted features. With the learning-based methods, the focus has shifted from manually defining image features to providing clean and correctly annotated data to the learning system. With the data-centric approach, however, new challenges arise.

In a clinical setting, when such algorithms are meant to be used as diagnostic assistance tools, the user has to be able to understand how the artificial intelligence (AI) system came up with the diagnosis. One key component in this regard is a measure of confidence of the AI system in its prediction. Such a measure is important to increase trust in the AI system, and it may improve clinical decision-making [1]. We will use the term “uncertainty estimation” for measures to evaluate model confidence. When the AI system provides a measure for its uncertainty, predictions with high uncertainties can be treated with extra care by medical experts. On the other hand, the human expert can better trust the prediction of an AI system where it reports low uncertainty. In this study, we review recent publications that have applied uncertainty estimation methods to medical image classification tasks. The area of uncertainty estimation in deep neural networks is an active research field, and the currently most popular methods have been proposed from 2016 onward. In the next section, we provide an overview of the most prominent methods for uncertainty estimation.

In the results section, we categorize the reviewed works by the uncertainty estimation method they apply. We provide a table that serves as an overview of all the included studies. In the last section, we discuss the most frequently used metrics for evaluating the benefit of uncertainty estimation and give an outlook of possible future research directions with a focus on human-machine collaboration.

### Technical Background

In a classification task, the neural network is supposed to predict how likely it is for a given input *x* to belong to class *y* out of a fixed number of possible classes. The output of the neural network can be interpreted as a probability distribution over all classes, with each individual value indicating how likely it is for the input to belong to the respective class.

In formula, the predictive distribution can be written as follows:

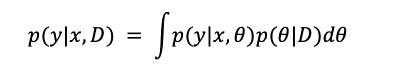



The predictive distribution given input *x* and training data *D* is described as the integral over the likelihood *p(y|x,θ)* with prior *p(θ|D)* computed over the model’s parameters *θ*. In deep neural networks, this integral cannot be computed analytically. Therefore, methods that try to capture uncertainty in neural networks try to approximate the predictive distribution.

Depending on the modeled uncertainty, the predictive uncertainty can be divided into aleatoric uncertainty and epistemic uncertainty. The aleatoric uncertainty describes the uncertainty inherent in the data, whereas the epistemic uncertainty captures the uncertainty of the model. The softmax output of a typical classification network is only able to capture aleatoric uncertainty [2].

### Methods for Uncertainty Estimation

Ovadia et al [3] compared several popular methods for uncertainty estimation. In this work, we name the methods that we discovered to be most popular and refer the reader to the respective works for a detailed description of the proposed methods. We categorize the methods into (1) model sampling, (2) single network methods, and (c) data augmentation.

#### Model Sampling

Sampling-based methods are easy to implement as they make use of existing network architectures. The 2 most popular methods are Monte Carlo dropout (MCDO) [4] and Deep Ensembles [5]. Both methods rely on several prediction runs of either an ensemble of multiple neural networks or a neural network with dropout layers to compute a predictive uncertainty.

#### Single Network Methods

The field of directly modifying the network architecture for improved uncertainty estimation is quite diverse. In the derivation of MCDO, the authors compare their approach to Gaussian processes (GPs). A GP is a method to estimate a distribution over functions [6] and can be applied to estimate uncertainties in neural networks.

Approaches that have been included in the comparison by Ovadia et al [3] include stochastic variational inference (SVI) [7] and temperature scaling (TS) [8]. SVI applies the concept of variational inference to deep neural networks, whereas TS works as a post hoc method. By applying a scaling factor to the network output, TS can improve network calibration. Another method worth mentioning is evidential deep learning (EDL) [9]. EDL fits a Dirichlet distribution to the network output to estimate the network’s uncertainty.

#### Data Augmentation

Comparable to sampling multiple models, one can also compute a distribution of predictions by running the network on different augmentations of the input data. Ayhan and Berens [10] propose such a method for improved aleatoric uncertainty estimation called test-time data augmentation (TTA).

## Methods

### Data Extraction

For the systematic review, we searched through Google Scholar, PubMed, IEEE Xplore, and ScienceDirect to identify relevant works that apply uncertainty estimation methods to medical image classification. We limited our search to works that have appeared between January 2016 and October 2021. As search terms, we used “uncertainty,” “uncertainty estimation,” “network calibration,” and “out-of-distribution detection,” and we combined them with the terms “medical images,” “medical image analysis,” and “medical image classification.”

### Selection Process

**Figure 1:**
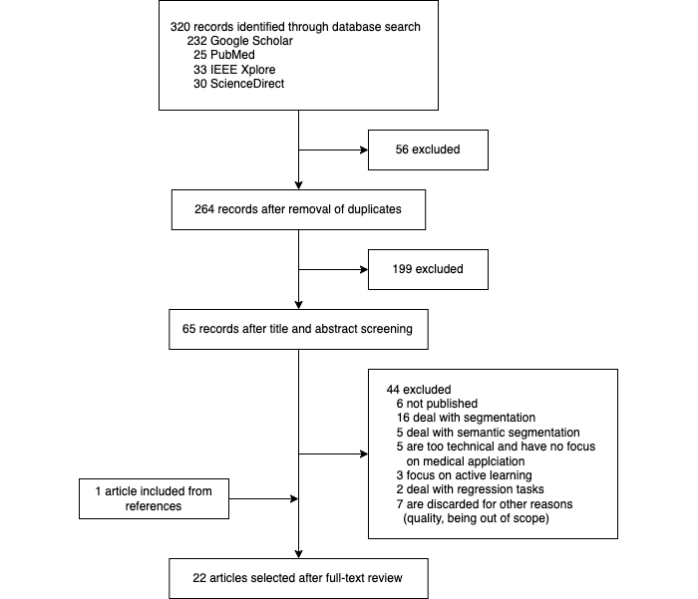
PRISMA (Preferred Reporting Items for Systematic reviews and Meta-Analyses) flow diagram.

The selection process was conducted according to the PRISMA (Preferred Reporting Items for Systematic Reviews and Meta-Analyses) guidelines [11]. We found 320 potentially relevant publications from the database search. During title and abstract screening, we discarded the majority of the works, as they either did not estimate uncertainties at all or dealt with other image analysis problems such as image segmentation. From the first screening round, 65 papers were selected for full-text analysis. During the full-text analysis, we discarded several other works, as they turned out to deal with other problems including semantic segmentation. Eventually, 22 papers were included in the review. Figure 1 visualizes the selection process.

## Results

### Paper Categorization

**Figure 2:**
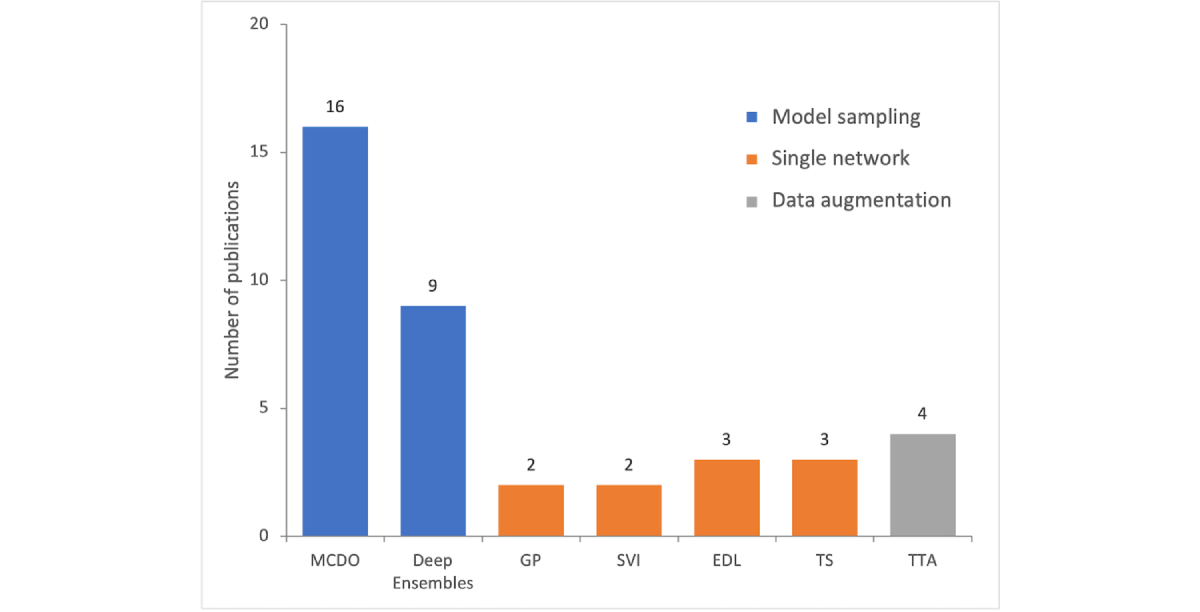
Number of publications that apply the respective uncertainty estimation method. EDL: evidential deep learning; GP: Gaussian process; MCDO: Monte Carlo dropout; SVI: stochastic variational inference; TS: temperature scaling; TTA: test-time data augmentation.

Figure 2 provides an overview of the applied methods in all of the reviewed works. Note that most included works apply more than 1 method for uncertainty estimation. We observed that the majority of works apply sampling-based methods (ie, MCDO and Deep Ensembles). In the category that we denoted as single network methods, all corresponding methods are almost equally represented. Lastly, 4 works that we included apply TTA to compute an uncertainty estimate.

**Figure 3:**
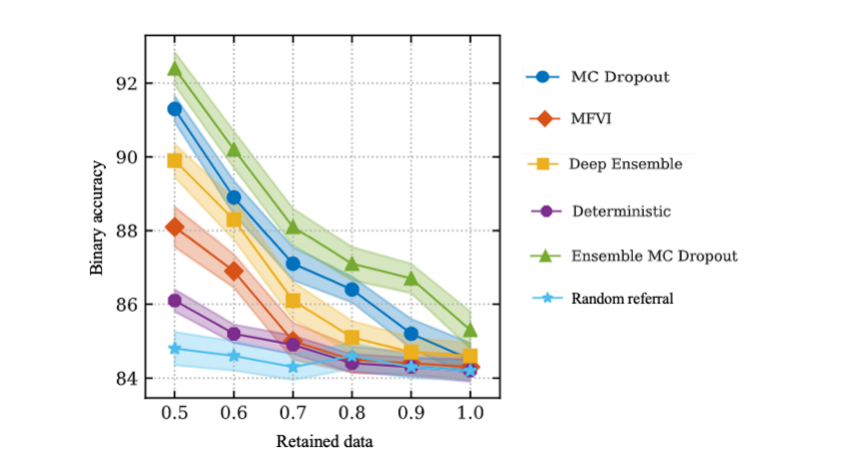
Retained data versus accuracy plot from Filos et al [2]. MFVI: mean field variational inference.

Most of the included works evaluate the applied methods by computing an uncertainty measure (mostly predictive variance or predictive entropy). This uncertainty measure is often used to generate retained data versus accuracy evaluations. Figure 3 shows an example of retained data versus accuracy plot from the study by Filos et al [2]. From the plot, it can be observed that when only the more certain samples are retained, accuracy on the retained data increases. The methods for uncertainty estimation are then ranked by how far they increase the accuracy on the retained data.

Some included works focus on network calibration and try to decrease the expected calibration error (ECE) within their experiments. Some other works use the computed uncertainty measure to detect out-of-distribution (OOD) samples. Table 1 provides an overview of all included works. In the following sections, we will briefly cover the content of each included study.

**Table 1:** Overview of the selected studies.

Methods	Organs or sickness	Sensor	Network architecture	Reported metrics	Data access	Code available	Reference
MCDO^a^, GP^b^	Diabetic retinopathy from fundus images	Camera	Custom CNNs^c^	Retained data or accuracy, uncertainty or density	Public (Kaggle competition)	Yes	Leibig et al [12]
MCDO, SVI^d^	Retina	Optical coherence tomography	ResNet-18	Predictive variance	Public	Yes	Laves et al [13]
MCDO	Skin cancer	Camera	VGG-16, ResNet-50, DenseNet-169	Uncertainty or density, retained data or accuracy, uncertainty, confusion matrix	Public	Yes	Mobiny et al [14]
MCDO	Brain	MRI^e^	Modified VGGNet	Reliability diagrams, AUROC^f^	Private	Yes	Herzog et al [15]
MCDO	Breast cancer	Mammography	VGG-19	Uncertainty, confusion matrix	Public	No	Caldéron-Ramírez et al [16]
MCDO, DUQ^g^	COVID-19	X-ray	WideResNet	Jensen-Shannon divergence	Public	No	Caldéron-Ramírez et al [17]
MCDO, Ensembles, MFVI^h^	Diabetic retinopathy from fundus images	Camera	VGG Variants	Retained data or accuracy, retained data or AUROC, ROC^i^	Public (Kaggle competition)	Yes	Filos et al [2]
MCDO, Ensembles, M-heads	Histopathological slides	Microscope	DenseNet	Retained data or AUROC	Public	No	Linmans et al [18]
MCDO, Ensembles, Mix-up	Histopathological slides	Microscope	ResNet-50	ECE^j^, AUROC, AUPRC^k^	Private	No	Thagaard et al [19]
MCDO, Ensembles	COVID-19, Histopathological slides (breast cancer)	CT^l^, microscope	ResNet-152-V2, Inception-V3, Inception-ResNet-V2	Predictive entropy, retained data or accuracy	Public	No	Yang and Fevens [20]
MCDO, Ensembles, TWD^m^	Skin cancer	Camera	ResNet-152, Inception- ResNet-V2, DenseNet-201, MobileNet-V2	Entropy, AUROC	Public (Kaggle competition, ISIC data set)	No	Abdar et al [21]
MCDO, Ensembles, others	Lung	X-ray	WideResNet	AUROC, AUPRC	Public	No	Berger et al [22]
GP	Diabetic retinopathy from fundus images	Camera	Inception-V3	AUROC	Public (Kaggle competition)	Yes	Toledo-Cortés et al [23]
EDL^n^ + Ensembles	Chest	X-ray	DenseNet-121	AUROC	Public	No	Ghesu et al [24]
EDL + MCDO	Breast cancer	Mammography	VGGNet	AUROC	Public + private	No	Tardy et al [25]
EDL	Chest, abdomen, and brain	X-ray, ultrasound, MRI	DenseNet-121	AUROC, coverage or F1 score, coverage or AUROC	Public	No	Ghesu et al [26]
TS^o^, MCDO	Polyp	Colonoscopy (camera)	ResNet-101, DenseNet-121	ECE, predictive entropy, predictive variance	Public + private	No	Carneiro et al [27]
TS, DCA^p^	Head CT, mammography, chest x-ray, histology	Multimodal	AlexNet, ResNet-50, DenseNet-121, SqueezeNet	ECE	Public	No	Liang et al [28]
TTA^q^	Diabetic retinopathy from fundus images	Camera	ResNet-50	Uncertainty or density, retained data or AUROC	Public (Kaggle competition)	Yes	Ayhan and Berens [10]
TTA, MCDO, MCBN^r^, Ensembles	Skin cancer	Camera	ResNet-50	ECE	Private (31,000 annotated images)	No	Jensen et al [29]
TTA + MCDO	Skin cancer	Camera	Efficient-Net-B0	Predictive entropy, predictive variance, Bhattacharya coefficient, retained data or accuracy	Public (ISIC data set)	No	Combalia et al [30]
TTA, TS, Ensembles	Diabetic retinopathy from fundus images	Camera	Modified ResNet	Reliability diagrams, AECE^s^, retained data or AUROC	Public (Kaggle competition)	Yes	Ayhan et al [31]

^a^MCDO: Monte Carlo dropout.

^b^GP: Gaussian process.

^c^CNN: convolutional neural network.

^d^SVI: stochastic variational inference.

^e^MRI: magnetic resonance imaging.

^f^AUROC: area under the receiver operating curve.

^g^DUQ: deterministic uncertainty quantification.

^h^MFVI: mean field variational inference.

^i^ROC: receiver operating curve.

^j^ECE: expected calibration error.

^k^AUPRC: area under the precision recall curve.

^l^CT: computed tomography.

^m^TWD: three-way decision theory.

^n^EDL: evidential deep learning.

^o^TS: temperature scaling.

^p^DCA: difference between confidence and accuracy.

^q^TTA: test-time data augmentation.

^r^MCBN: Monte-Carlo batch norm.

^s^AECE: adaptive expected calibration error.

### Sampling-Based Methods

The first work that we have included is the study by Leibig et al [12], which applies MCDO to the task of diabetic retinopathy classification. To evaluate the impact of the applied uncertainty estimation method, the authors report retained data versus accuracy curves. This means that a fraction of uncertain predictions is discarded, and it is evaluated how discarding uncertain samples can improve the accuracy on the test data set. The results show that discarding 20% or more of the most uncertain samples can notably improve the accuracy of the neural network. In their work, the authors compare the performance of MCDO to an alternatively implemented GP and find that MCDO leads to better accuracies on the retained data versus accuracy evaluations.

Laves et al [13] apply MCDO and SVI to retina scans observed through optical coherence tomography. The authors show that both methods lead to higher standard deviations on false-positive predictions compared to true positive predictions. This indicates that the standard deviations can be used to refer predictions with high uncertainty to human experts to improve the classification accuracy.

Mobiny et al [14] estimate uncertainties using MCDO with different types of networks including VGGNet [32], ResNet [33], and DenseNet [34] on dermoscopic images of 8 different skin lesion types. Similar to Leibig et al [12], the authors report retained data versus accuracy curves and show that the accuracy can be increased when referring a fraction of uncertain samples to a medical expert. As a measure for uncertainty, the normalized predictive entropy is computed. As an additional metric, the authors also compute an uncertainty-related confusion matrix that includes the numbers of correct-certain, correct-uncertain, incorrect-certain, and incorrect-uncertain predictions. The respective numbers vary when the uncertainty threshold is changed. One possible goal with this evaluation is to decrease the number of incorrect-certain predictions as much as possible.

Another work by Herzog et al [15] applies MCDO to the classification of brain magnetic resonance imaging (MRI) images. The goal of their work is to infer patient-level diagnostics from the predictions from multiple images. Therefore, the authors compute a variety of 5 uncertainty measures per image. To draw conclusions on a patient level, the authors run another neural network that processes the uncertainties of all images belonging to one patient.

In two other published works, Caldéron-Ramírez et al [16,17] apply MCDO to the tasks of breast cancer classification from mammography images and to COVID-19 classification from chest x-ray scans. Unfortunately, even among the two works, the authors report different metrics, which prevents comparing the results. In the breast cancer classification task, the authors use a metric called uncertainty balanced accuracy, which builds up on the uncertainty-related confusion matrix also used by Mobiny et al [14]. In the work related to COVID-19 detection, the authors report the Jensen-Shannon divergence as an uncertainty measure, which we did not encounter in any of the other reviewed works.

Another set of studies compared MCDO to Deep Ensembles (further simply denoted as Ensembles) and partly to other methods. Filos et al [2] compare MCDO to Ensembles and mean field variational inference (MFVI), which is a variation of SVI, and apply it to the task of diabetic retinopathy classification. In addition to comparing MCDO and Ensembles individually, they also combine both approaches and include the combination in the evaluation, denoted as “Ensemble MCDO.” As neural network architecture, the authors use variants of VGGNet [32]. The retained data versus accuracy plots show that “Ensemble MCDO” leads to the best performance, followed by MCDO and Ensembles applied individually. MFVI did not achieve the same performance as the sampling-based methods.

Linmans et al [18] perform uncertainty estimation on the publicly available Camelyon data sets for breast cancer detection on histopathological slides. The authors propose a new method for uncertainty estimation called “M-heads,” which adds multiple output heads to the convolutional neural network (CNN). They compare their method to the MCDO and Ensembles of 5 and 10 networks, respectively. From the different evaluations, the confidence versus accuracy plot shows that accuracy increases when only keeping predictions with high confidence. The methods rank from M-heads performing best, followed by the Ensembles of 5 and 10 networks. In the reported results, MCDO does not perform better than the vanilla softmax output.

Thagaard et al [19] apply Ensembles and MCDO to private data sets of histopathological slides for breast cancer detection. In their work, the authors focus on OOD detection while analyzing combinations of different internal data sets. Concerning the comparison of the uncertainty estimation methods, the ECE is calculated on 3 different data sets. For all 3 data sets, the Ensemble of 5 ResNet-50 networks reaches the best ECE scores.

In another work, Yang and Fevens [20] apply MCDO, Ensembles, and a combination of both to several publicly available data sets. The modalities include COVID-19 classification from x-ray scans, brain tumor classification from MRI images, and breast cancer detection from histopathological slides. On the histopathological images, the authors present retained data versus accuracy plots. For the reported accuracies, the Ensemble MCDO approach with 5 Inception-ResNet networks leads to the best results.

Abdar et al [21] apply MCDO, Ensembles, and Ensemble MCDO to skin cancer classification from dermoscopic images. The authors report entropies and standard deviations of the applied methods for 4 different network architectures on 2 different publicly available data sets. From the reported values, the authors conclude that the Ensembles overall perform best. In an additional setup, the authors combine 2 uncertainty estimation methods (Ensembles and Ensembles MCDO) in a decision tree that they refer to as 3-way decision theory.

In another work, Berger et al [22] evaluate confidence-based OOD detection on x-ray scans of lung diseases. The authors compare MCDO, Ensembles, and specific methods for OOD detection, including a method based on Mahalanobis distance and the “out-of-distribution detector for neural networks” [35]. In their experiments, the authors find that the OOD detector for neural networks leads to the best results for OOD detection with respect to the area under the receiver operating curve (AUROC) and area under the precision recall curve (AUPRC) values.

### Single Network Methods

After having covered several works that focus on sampling-based uncertainty estimation methods, we will now look into works that directly apply to the network’s classification output to estimate uncertainties. One example is the work by Toledo-Cortés et al [23] that applies a GP to the output of their implemented Inception-V3 network [36]. Similar to Laves et al [13], the authors report standard deviations on true positive and false positive predictions. Since the standard deviations for both cases are quite similar, it must be concluded that the applied GP is not well suited for a useful uncertainty estimation.

A set of other works applies EDL to estimate uncertainties. In their first work, Ghesu et al [24] work with x-ray scans of the chest and later extend their approach to ultrasound images of the abdomen and MRI images of the brain [26]. The results show that discarding a fraction of the most uncertain predictions can notably improve the AUROC score averaged over different x-ray classification tasks.

Comparably, Tardy et al [25] apply EDL to the task of breast cancer classification from mammography images. The authors also report improved AUROC and AUPRC values when discarding a fraction of uncertain samples.

Two works that we have included apply TS to medical image classification tasks. Carneiro et al [27] combine TS and MCDO to compute a calibrated confidence measure as well as an uncertainty measure in the form of predictive entropy and predictive variance. The authors evaluate the methods on 2 different cohorts of colonoscopy images with respect to a 5-class polyp classification task. The reported ECE and accuracy values show that the DenseNet-121 architecture with both MCDO and TS leads to the best results.

Liang et al [28] present a new approach for network calibration in the form of an auxiliary loss term called “difference between confidence and accuracy” (DCA) that can be integrated into an existing CNN training procedure. The authors compare their approach to TS and uncalibrated networks on different medical data sets with several different network architectures. The results show that in most cases, DCA produces the best ECE values. It is also shown that depending on the data set and model architecture, TS does not always improve the expected calibration error.

### Test-Time Data Augmentation (TTA)

The concept of TTA is introduced by Ayhan and Behrens [10], where it is applied to the task of diabetic retinopathy from fundus images. The authors apply 128 different augmentations, ranging from cropping and resizing to different color augmentations. As measure for uncertainty, the interquartile range of the predictions is computed. Similar to Leibig et al [12], the authors report retained data versus AUROC curves and show that the AUROC values improve when referring uncertain samples to a medical expert.

Another work by Jensen et al [29] focuses on evaluating interrater agreement on dermoscopic images of different skin lesions. In the experiment, multiple experts have provided labels for the respective images, and the labels for each sample can vary across experts. Therefore, the approaches of label fusion and label sampling are compared for training the neural network. These approaches are combined with methods that estimate uncertainties to evaluate the influence on the network’s calibration of the combined methods. It is shown that in the specific experimental setting, the combination of label sampling and TTA leads to the highest classification accuracies among all data splits.

Combalia et al [30], also working with dermoscopic images, combine TTA and MCDO to evaluate aleatoric as well as epistemic uncertainties. In their experiments, the authors show that the combination of both methods leads to the best results for OOD detection as well as on the retained data versus accuracy evaluation. For the evaluations, 100 forward passes through the network are performed with either TTA or MCDO or both methods combined. The uncertainties are quantified by computing the predictive entropy, the predictive variance, and additionally, the Bhattacharyya coefficient [30].

In a follow-up of their original work, Ayhan et al [31] extend their experiments on diabetic retinopathy classification by other uncertainty estimation methods. Besides TTA, the authors also include TS and an ensemble of 3 modified ResNet networks. To compare the results, the authors compute The Adaptive Expected Calibration Error [37]. In terms of Adaptive Expected Calibration Error, the median probability of 128 forward passes with different data augmentations leads to the best calibrated results. On the retained data versus AUROC curves, TTA and Deep Ensembles perform equally well. The experiments on a different cohort of fundus images show that TS generalizes worse to new data compared to TTA and Deep Ensembles.

## Discussion

Through the reviewed publications, we gained an overview of which methods for uncertainty estimation are most frequently used in the field of medical image classification. We found that the sampling-based methods MCDO and Deep Ensembles are the most frequently applied methods. With the sampling-based approaches, it is possible to compute a distribution of predictions and from there determine an uncertainty measure, usually either in the form of predictive entropy or predictive variance. These measures help to identify samples where the neural network is uncertain about its predictions.

In addition to the sampling-based uncertainty evaluations, we also observed evaluations that analyze the calibration of the neural network. The calibration evaluations in terms of reliability diagrams and ECE are used to determine if the neural network’s output probabilities represent the actual likelihood of the prediction being correct. In the original paper on neural network calibration [8], the authors claim that most modern CNNs are not well calibrated and produce overconfident predictions. In this review, we saw that several methods including TS and TTA can be applied to improve calibration [31].

Another observation we made is that combining uncertainty estimation methods can improve the results. This holds for combinations of Ensembles and MCDO [2,20,21], TS and MCDO [27], or TTA and MCDO [30].

By presenting retained data versus accuracy curves, several works [2,10,12,14,20,26,30] show that discarding uncertain predictions leads to an improved accuracy of the neural network on the remaining samples. This insight holds for all 3 categories of uncertainty estimation methods that we denoted as (1) model sampling, (2) single network methods, and (3) data augmentation. An important message from this observation is that uncertainty estimation can be used as a tool to improve the collaboration between AI systems and human experts. Thus far, all studies were performed in very artificial settings. Future work should therefore analyze the performance improvement of a collaboration between an uncertainty-aware AI system and human experts in scenarios that are closer to real-life situations in clinics.

## Abbreviations

AI: artificial intelligence
AUPRC: area under the precision recall curve
AUROC: area under the receiver operating curve
CNN: convolutional neural network
DCA: difference between confidence and accuracy
ECE: expected calibration error
EDL: evidential deep learning
GP: Gaussian process
MCDO: Monte Carlo dropout
MFVI: mean field variational inference
MRI: magnetic resonance imaging
OOD: out-of-distribution
PRISMA: Preferred Reporting Items for Systematic Reviews and Meta-Analyses
SVI: stochastic variational inference
TS: temperature scaling
TTA: test-time data augmentation
